# Psychophysiological Adaptations to Pilates Training in Overweight and Obese Individuals: A Topical Review

**DOI:** 10.3390/diseases10040071

**Published:** 2022-09-29

**Authors:** Alexios Batrakoulis

**Affiliations:** Department of Physical Education and Sport Science, University of Thessaly, 42100 Trikala, Greece; abatrakoulis@uth.gr; Tel.: +30-24310-47018

**Keywords:** Pilates, exercise, overweight, obesity, physiological responses, psychological responses

## Abstract

The prevalence of overweightness and obesity has been documented as a major public health issue since it has increased at an alarming rate worldwide. Structured physical exercise programs have been reported as an essential strategy for preventing, managing, and treating obesity, inducing critical improvements in various physiological and psychological markers. However, it is unclear whether Pilates training can elicit positive changes in body composition, physical fitness, cardiometabolic health, and well-being among overweight and obese populations. The purpose of this topical review was to catalog studies investigating the physiological and psychological adaptations to Pilates training in order to identify what outcomes have been assessed, the research methods used, and the results. The inclusion/exclusion criteria were met by 14 published articles involving 582 participants (83% female) who were overweight or obese. The present topical review on Pilates training-induced adaptations shows that this widely used exercise type can significantly improve the majority of the selected indicators. These beneficial changes are frequently focused on anthropometric parameters, body composition, glucose, and lipid metabolism, as well as blood pressure in sedentary overweight or obese women. Specialized equipment-based Pilates interventions and trials investigating various mental health indices were limited. Further research is warranted in this area, emphasizing the Pilates training configuration and potential mechanisms behind positive alterations in several psychophysiological markers through large-scale randomized controlled trials with superior methodological quality, implementing long-term interventions in various populations that are overweight and obese.

## 1. Introduction

### 1.1. The Obesity Epidemic

Obesity is a complex chronic disease defined as an excessive body fat accumulation predisposing to impaired metabolic health, which is responsible for developing several obesity-related chronic diseases [1]. The prevalence of overweightness and obesity has increased at a truly alarming rate recently. Specifically, 52% of the world’s adult population are overweight or obese, and the adult obesity rates have tripled since 1975 [2]. The cost of obesity-related illness is estimated to be US $2.0 trillion every year starting from 2025, impacting the economic climate and public health globally [3]. Obesity is a major health issue with an intersectoral etiology with costly implications for society, resulting in several cardiometabolic complications through numerous physiological and psychological abnormalities [4]. Noticeably, adults with overweightness and obesity are likely to demonstrate insufficient physical activity [5], impaired mental health [6], and poor functional capacity [7]. Cardiorespiratory and musculoskeletal fitness as well as metabolic health appear to be critical factors that limit these populations from engaging in a structured exercise program [8]. Given that weight maintenance has been reported as a more challenging and vital goal than weight loss among individuals with an unhealthy body mass [9], numerous physiological and psychological health factors may play a crucial role in this long-term goal [10]. Interestingly, only one in five of those who try to lose weight succeed and only those who maintained a 10% loss within a year. In addition, regular engagement in high physical activity levels seems to be the optimal strategy for weight loss maintenance in populations that are overweight and obese [11].

### 1.2. Exercise and Obesity

Physical exercise has been widely recommended as an essential part of a multicomponent strategy against the obesity epidemic, promoting significant improvements in metabolic health, physical fitness, and well-being in previously inactive overweight and obese individuals [12,13,14,15]. However, only 31% of adults are classified as sufficiently physically active worldwide [16], and thus physical inactivity has been documented as a major global public health issue of the 21st century since it is associated with the most common lifestyle-related chronic conditions [17]. As such, several types of exercise for overweight and obesity individuals are currently highlighted as some of the most attractive health and fitness trends [18]. Specifically, the latest exercise prescription guidelines for overweight and obese individuals underline the importance of combined aerobic and resistance training either in a single session or as separate weekly sessions [14]. In addition, recent data show that combined training is the most effective exercise mode for improving a wide spectrum of cardiometabolic health-related parameters in populations with unhealthy weight [15]. However, such a time-consuming exercise strategy [19] demonstrates high dropout and low adherence rates among adults with overweightness and obesity [20]. On the other hand, high-intensity interval training appears to be a time-efficient [21,22] and popular [23] exercise type, but such a demanding approach is not clearly feasible and attractive to populations that are overweight and obese [24] given that lack of time has been reported as a major barrier to exercise among adults [25,26,27].

### 1.3. Pilates Training and Obesity

**Figure 1:**
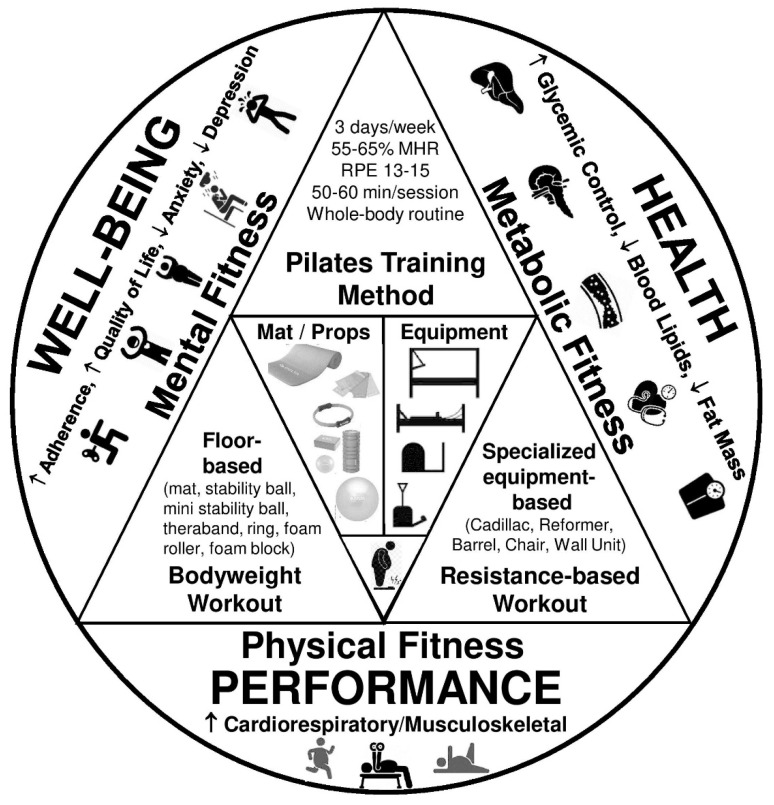
The psychophysiological effects of Pilates training. MHR: maximum heart rate; RPE: rating of perceived exertion. [32,33,34,35,36,38,39,52].

Pilates training is a mind–body exercise form founded by Joseph Pilates during the 1920s [28]. This alternative type of exercise emphasizes muscle control, posture, and breathing, applying floor- or specialized equipment-based exercises found in other forms of resistance training enhancing core stability, muscular strength and endurance, flexibility, and balance [29]. Special equipment is designed as spring-based resistance machines using a small bed frame with a sliding platform hooked up to a system of springs, ropes, and pulleys. Pilates training exercises are repeated in sets that strategically activate the muscles without exhausting them. Such an exercise approach appears to be user-friendly and safe for inexperienced trainees [29]. Most relevant studies focus on the efficacy of Pilates training on rehabilitation (e.g., chronic low back pain, postural, and movement dysfunction) [30], physical performance in healthy adults [31], as well as health and fitness benefits in older adults [32,33]. Therefore, Pilates training has been defined as a therapeutic or preventive exercise intervention [29]. On the other hand, only few studies investigated the role of Pilates training in cardiorespiratory fitness [34], body composition [35,36], and cardiometabolic [37,38] and mental health [39] in various populations. It is worth noticing that Pilates training mainly attracts physically inactive, middle-aged women who are primarily interested in improving physical fitness (e.g., body posture and flexibility) and secondarily in rehabilitation (e.g., pain and disability reduction) while demonstrating some pain or musculoskeletal discomfort [40]. Thus, Pilates training is frequently prescribed to individuals suffering from musculoskeletal health issues and poor functional capacity due to its emphasis on the activation of trunk muscles that enhance lumbar spine stability while promoting reduced pain and disability [41]. Notably, Pilates training, as a physical exercise method, has differentiated in order to align with recent scientific evidence, aiming to serve various populations in sports, fitness, and clinical settings [42]. Thus, it is considered a widely used therapeutic or preventive exercise intervention, although it is not currently included in top health and fitness trends worldwide according to the latest regional [43,44,45,46] and global reports [47]. Recently published data on the effects of such an alternative type of exercise on body composition in overweight and obese individuals showed remarkable improvements [35]. However, available data are limited concerning the effectiveness of Pilates training on various physical and mental fitness parameters in these populations. Interestingly, recent studies indicate inconsistent results without a clear consensus [48,49,50], and comparative studies are scarce [51,52]. Figure 1 summarizes the effects of Pilates training on cardiometabolic health, physical performance, and well-being in several populations [32,33,34,35,36,38,39,52].

The purpose of this brief review was to abridge the research methods used and the main findings displayed in Pilates-based training studies where selected physiological (e.g., anthropometric, body composition, physical fitness, and cardiometabolic health-related indicators) and psychological outcomes (e.g., adherence, exercise enjoyment, depression, anxiety, and quality of life) were investigated. Such a review article may circulate the main outcomes on psychophysiological adaptations to Pilates training in overweight and obese individuals, aiming to detect research issues, considerations, and gaps in the literature. Given the serious lack of evidence on physiological and psychological responses to Pilates training method, all relevant studies were collected, regardless of their methodology, in order to distribute a wide-ranging viewpoint on the current evidence. The present brief review addressed the general question: ‘What is known from published research about selected psychophysiological responses to numerous Pilates-based training interventions in individuals with overweight and obesity?’

## 2. Materials and Methods

### 2.1. Literature Search Strategy

The PubMed and MEDLINE databases were used to retrieve relevant articles from inception up to 1 August 2022 after a systematic electronic search by the author (A.B.) and a research assistant (P.B.). The search algorithm used the following terms: overweight, obese, obesity, Pilates. The complete search strategy is provided in the online supplemental material (Table S1). Reference lists from articles were searched to retrieve additional potentially eligible articles.

### 2.2. Eligibility Criteria

Studies were considered eligible for inclusion if the following criteria were met: (1) participants were individuals of any age with no diagnosed comorbidities, signs/symptoms of any noncommunicable disease or eating disorders, pregnancy, and with a body mass index (BMI) ≥ 25 kg/m^2^; (2) included studies employed an intervention of various forms of Pilates; and (3) examined at least one of the following physiological and psychological outcomes in humans: body weight, BMI, body fat percentage, lean body mass, waist circumference, blood glucose, lipid profile, blood pressure, cardiorespiratory fitness, muscular strength, muscular endurance, flexibility, balance, functional capacity, adherence, exercise enjoyment, depression, anxiety, and quality of life. All studies were required to be written in English and published in a refereed journal from inception up to 1 August 2022. 

As for the exclusive criteria, studies were not considered eligible for inclusion if the following criteria were met: (1) studies recruited a mixed sample of individuals with overweightness/obesity and other chronic diseases per intervention arm; (2) articles where the effects of Pilates training intervention cannot be isolated because it was involved as part of a multicomponent exercise intervention (e.g., a training program consisting of Pilates and aerobic or resistance training); (3) articles that did not evaluate the selected outcome measures; (4) studies published in languages other than English; and (5) studies that had not undergone full peer review (e.g., conference proceedings, posters, published abstracts, lay articles, proposed studies, dissertations, theses, reviews, commentaries, and debates).

### 2.3. Study Selection

**Figure 2:**
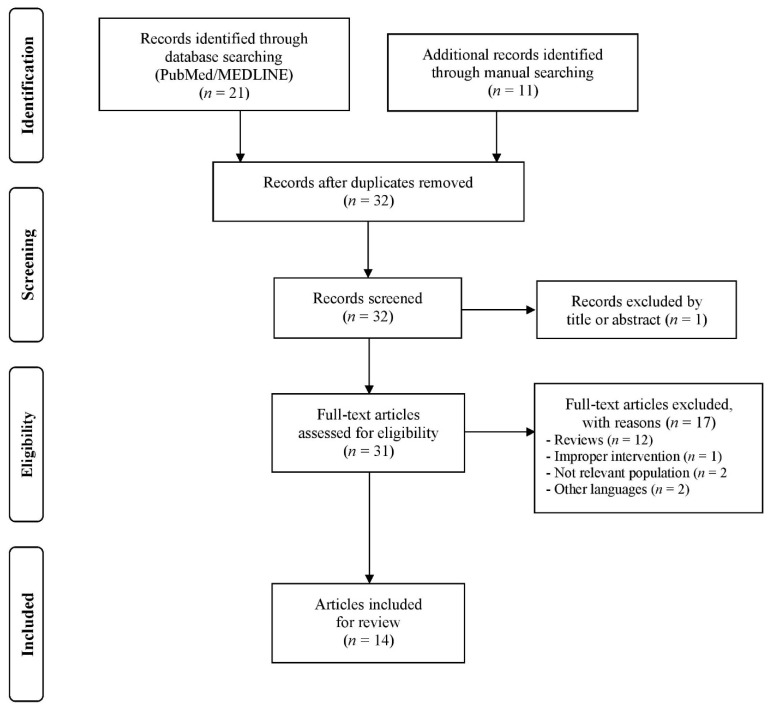
Flowchart of the systematic literature search.

The author (A.B.) and a research assistant (J.W.) independently screened the titles and abstracts of potentially eligible studies and downloaded the full texts of the remaining articles to assess their eligibility. Any discrepancies between the two assessors were resolved by discussion and consultation with a research fellow (L.T.). EndNote X9 (Clarivate Analytics, Philadelphia, PA, USA) literature management software was used to manage the literature search records. The flow diagram is illustrated in Figure 2, showing the literature search and selection process in detail.

### 2.4. Data Extraction

The author (A.B.) and a research assistant (J.W.) independently extracted data using Microsoft Excel. Any disagreements were resolved by consensus. In case of insufficient information, the authors of the included studies were contacted via email for missing values where required. Data extraction included first author, year of publication, country, intervention duration, sample size, participant demographics (e.g., gender, mean age, and activity level), study design, Pilates training classification (mat/props or equipment), Pilates training intervention details (frequency, intensity, time, and type), and critical psychophysiological outcome measures and findings reported from each eligible study, as shown in Table 1. 

## 3. Results

### 3.1. Articles Retrieved

The electronic search returned 32 articles. After screening titles, abstracts, and full texts, 14 eligible studies were included in this review (Figure 2). Relevant data extracted from each article are presented in Table 1.

### 3.2. Article Characteristics

Articles were published from 2006 to 2020 (2006–2015: *n* = 5, 2016–2020: *n* = 9), and research was conducted in eight countries. There was a total sample of 582 participants (83% female) with overweightness or obesity across all studies. One (7%) article reported on the investigation of psychological measures as a primary outcome, and all articles reported on investigations of chronic responses (≥2 weeks) to Pilates training protocols. Training studies lasted from 4 to 24 weeks in duration (≤12 weeks: 86%; >12 weeks: 14%), with exercise session frequency ranging from three to five times per week, and used quantitative methods. Articles reported on studies that implemented randomized controlled trials (*n* = 12, 86%) or controlled trials (*n* = 2, 14%). Articles reported on studies that assigned supervised (*n* = 13, 93%) or semi-supervised (*n* = 1, 7%) Pilates training interventions. Articles reported on studies that were conducted in a lab-based (*n* = 2, 14%) or field-based (*n* = 12, 86%) environment.

### 3.3. Exercise Protocols

Of the 14 Pilates training protocols applied in the reviewed studies, 12 (86%) were classified as floor-based (matwork with props), and 2 (14%) were classified as specialized equipment-based (Cadillac and Reformer). The authors did not present details of the Pilates interventions considering the prescribed training parameters (e.g., frequency, intensity, time, and type). The most frequently reported Pilates training protocol was a floor-based protocol (matwork, 3 sessions/week, 60 min/session). 

## 4. Discussion

### 4.1. Summary of Main Results

Through a mini review, 14 articles were systematically detected that published psychophysiological outcomes associated with Pilates training in previously inactive, overweight, and obese individuals. The large majority (93%) of eligible studies included in this review examined floor-based forms of Pilates training (matwork with props) in a supervised (86%) and lab-based setting (14%). The finding that 57% (8/14) of the eligible articles were published between 2017 and 2020, while 5 studies (35%) were published in 2020, underlines the novelty of this particular emerging research topic. The present brief review abridged the current literature to support further discussion of the questions, reflections, and gaps that need to be considered in future studies. Overall, Pilates-based training interventions appear to be a beneficial exercise approach for overweight and obese individuals, mostly seeking to improve body composition and physical fitness. As for cardiometabolic and mental health-related indices, no strong evidence was found across all eligible studies investigating these particular adaptations in this cohort. Additionally, the lack of data in all eligible studies regarding adverse events does not help identify how safe and feasible Pilates-based training programs are in a real-world gym setting for this population.

### 4.2. Body Composition

Anthropometric parameters and body composition have been the most frequently studied physiological outcomes in the Pilates training literature for overweight and obese individuals. On the other hand, there is currently a scarcity of research to test whether psychological outcomes can be obtained by this population following various Pilates training protocols. However, Pilates training programs have been widely established in the health and fitness industry worldwide [66,67]. The main results in the present topical review show that participants experienced meaningful improvements in body weight, BMI, body fat, and waist size but not in lean body mass and bone metabolism markers. This is an important finding highlighting the effectiveness of less vigorous, but more time-consuming, Pilates-based exercise protocols on weight management. Taking this into account, the commonly used weekly exercise volume of 180 min (3 times per week, 60 min per session) across all studies included in the present review seems to be a key training parameter providing an important energy expenditure that is considered a critical factor for inducing beneficial alterations in energy balance promoting weight and fat loss in sedentary, non-dieting overweight and obese individuals [14,68]. This particular finding coincides with recent evidence showing that the average exercise intervention for individuals with overweight and obesity was of moderate to vigorous intensity, 4 times per week, 50 min per session [35,69]. Furthermore, since Pilates training is defined as an alternative resistance-based exercise mode incorporating various whole-body movements [70], it appears possible to increase muscular performance [71], contributing to body composition improvements. It was documented that a single Pilates training session lasting 30–45 min may induce beneficial alterations in energy expenditure, resulting in body weight and fat reductions [72]. In addition, such an exercise mode appears to be beneficial for increasing physical–functional performance associated with body composition improvements [73,74]. However, some review studies showed that Pilates training might not be an efficient type of exercise for improving various body composition indices in various populations [52,75,76], providing inconsistent results compared to the present work and a recent review focusing on overweight and obese adults [35]. 

### 4.3. Cardiometabolic Health

Given that raised cardiovascular risk factors are common among overweight and obese individuals [4,5], an in-depth investigation of the effectiveness of Pilates training on various physiological health indicators may be vital for identifying the role of this mind-body exercise type in the prevention, management, and treatment of overweightness and obesity. It has been well documented that this population is predisposed to the development of cardiometabolic diseases, mainly due to insufficient physical activity, an increase in the concentration of visceral adipose tissue, chronic inflammation, and insulin resistance, and increased oxidative stress [4]. Recent data show that various exercise modes promote positive alterations in various physiological outcome measures related to glucose and lipid metabolism, blood pressure, and physical fitness in populations with an unhealthy body mass [15]. However, inconsistent results were found in this mini-review regarding the effects of various Pilates training interventions on key cardiometabolic-health-related markers in these populations. In general, the efficacy of such an alternative resistance-based exercise mode on cardiovascular risk factors in previously inactive overweight and obese individuals has been poorly investigated, and there is no robust evidence concerning the comparative efficacy of different exercise types in this research area [70,71]. However, the main results presented here are in agreement with those reported for overweight/obese individuals with comorbidities after following various Pilates-based exercise interventions [37,49,77,78,79]. 

### 4.4. Physical Fitness

Pilates training is not a type of cardiovascular exercise; however, it may elevate cardiorespiratory fitness levels in previously inactive adults compared to controls [34]. In general, it is a neuromuscular training modality characterized by repeated movements utilizing various types of resistance to activate all muscles with an emphasis on the torso and without reaching muscle exhaustion [29]. Therefore, such an exercise approach promotes improvements in muscular endurance, core stability, balance, flexibility, and posture [31] while reducing exercisers’ odds of becoming injured [30]. Taking this into account, it seems that this mind–body exercise mode may lower the risk for various musculoskeletal and joint injuries [30] that are common among overweight and obese individuals due to their poor functional capacity [7]. Pilates training protocols appear to be effective for elevating essential motor skills resulting in a healthy spine and elevated performance in activities of daily living. This fact is important, considering that populations with unhealthy weight are likely to demonstrate postural issues, movement dysfunction, and several physical limitations [80,81,82]. It is of note that Pilates-based training programs demonstrate positive effects on physical–functional performance in both normal weight and overweight older adults [32,33,51]. Hence, such an alternative muscle-strengthening activity may provide an inclusive environment and exceptional improvements in functional task outcomes [49]. However, in the present review, only one study investigated flexibility [53] and maximal aerobic capacity [60] in this cohort, highlighting the gap in the literature concerning strong evidence related to the efficacy of Pilates training on physical and motor fitness parameters in individuals with overweightness and obesity. On the other hand, a few studies investigating the Pilates training method have shown that it can be an effective exercise mode for increasing functional capacity, flexibility, balance, posture, and muscular performance in various healthy and clinical populations [74,83,84]. Taking these observations into consideration, it does not seem clear the degree of improvement in a wide spectrum of musculoskeletal fitness parameters following Pilates training, as previously reported for other popular neuromuscular exercise types in these populations [85,86].

### 4.5. Mental Health

Considering that psychological health-related disorders are frequently present among individuals with an unhealthy weight [12,87,88,89,90], an in-depth investigation of the effectiveness of exercise training, including Pilates-based training protocols on several mental health indicators, may be vital for assessing the role of various physical exercise types in the prevention, management, and treatment of overweightness and obesity [12,87]. Recent evidence shows that regular exercise can lead to positive alterations in several mental health markers, such as quality of life and vitality [12,87]. However, no significant changes were found in anxiety and depression for overweight and obese individuals participating in various types of exercise [12]. Interestingly, the role of regular exercise in key psychological health markers in individuals with an unhealthy body mass has been poorly investigated, and there are no robust data concerning the comparative efficacy of different training modalities on this particular research topic [70]. Noticeably, Pilates training appears as an effective exercise strategy for elevating the quality of life and mood in the elderly [51], who are very likely to demonstrate an abnormal BMI [1]. In the present topical review, only one eligible article studied the effects of Pilates and aerobic training on anxiety, depression, and quality of life, demonstrating that both types of exercise are beneficial for improving these key psychological health markers in this population [64]. However, given the serious lack of data regarding the Pilates training-induced improvements in psychological health, it remains unclear to what degree such an alternative exercise mode is able to promote meaningful changes in psychometrics.

Of particular importance is that the large majority of eligible studies (86%) reported low dropout rates ranging from 0% to 14%. Given that 93% of studies included in this review implemented a supervised Pilates training intervention in a field-based environment, it seems that supervision by qualified instructors may play a key role in adherence to structured exercise. This is an important observation since lack of time has been widely documented as the most critical exercise barrier among adults [25,26,27], while individuals with overweightness and obesity demonstrate significantly high attrition and low compliance rates when engaging in various exercise types [20]. However, 86% of eligible studies implemented short-term (≤12 weeks) interventions, and thus low dropout rates observed in these studies cannot be considered strong evidence for the impact of Pilates training protocols on exercise adherence. Interestingly, muscle-strengthening activities are recommended to overweight and obese individuals twice per week as a critical piece of the exercise programming puzzle [14,90], and therefore Pilates training may be considered as an alternative resistance-based training modality for this population. 

### 4.6. Future Research

The small number of available trials delivers limited data concerning the efficacy of Pilates training on selected physiological and mostly psychological endpoints in overweight and obese individuals. Overall, the feasibility of Pilates training as a common type of exercise for these populations is still unclear. More rigorous research is needed to detect whether this mind–body exercise strategy can play a valuable role in the exercise programming puzzle adapted for previously inactive individuals with overweightness and obesity. However, from a physiological standpoint, the current evidence supports the viability of Pilates training as an alternative resistance-based exercise approach since it demonstrates positive alterations in several biomarkers in this population. Further research is needed on this topic, emphasizing examining the dose–response relationship of long-term Pilates training interventions (>12 weeks) and cardiometabolic as well as mental health-related indicators for overweightness and obese people in a real-world gym setting. Additionally, the potential mechanisms behind beneficial changes in several psychophysiological health markers should be explored in the future through larger samples and high-quality randomized controlled trials conducted in real-world conditions.

Since data considering the efficacy of Pilates training on key psychosocial indices such as anxiety, depression, mood state, quality of life, exercise enjoyment, affect valence, and adherence are currently limited for this population, the outcomes briefly summarized here may lead researchers to study Pilates training-based interventions promoting favorable changes in selected psychophysiological markers in sedentary overweight and obese individuals. Such a mind–body exercise approach may be a useful strategy to determine how to participate the vast majority of the adult population globally in structured and adapted Pilates training programs characterized by supervision, progression, and variety. Moreover, additional randomized controlled trials with an emphasis on attrition and compliance to such an alternative exercise mode are warranted, given that Pilates training has been reported as one of the most popular fitness services among health club/gym/studio members and especially women [40,66,67]. In taking this into consideration, further trials should investigate whether Pilates training provides an inclusive environment in order to identify if this type of exercise is effective, feasible, and attractive to men. Lastly, the present topical review clearly shows that Pilates training configuration should be a priority in future research attempts, aiming to provide practitioners with insights into various training parameters. More specifically, details regarding exercise intensity, volume, progression, and overload were limited across all eligible studies included in this review.

### 4.7. Strengths and Limitations

The present topical review has numerous strengths: (i) the rigorous study selection process; (ii) the classification (floor- or specialized equipment-based) of Pilates training protocols administered across all studies; (iii) the provision of reasonable suggestions for further research in this popular topic; and (iv) the summary as well as distribution of important research findings regarding the psychophysiological adaptations to Pilates training in the masses affected by overweight or obesity worldwide. However, this study has several limitations that should also be mentioned. Surely, it does not evaluate the quality of current data in the primary research studies, and therefore this mini-review presents a narrative clarification of existing evidence without addressing the synthesis of available data. Such a review study is primarily focused on a variety of study designs and methodologies than a systematic review, which is likely to emphasize a comprehensive analysis of a smaller number of randomized control trials.

## 5. Conclusions

Pilates training is an alternative resistance-based exercise strategy that induces psychophysiological adaptations associated with improved anthropometric parameters, body composition, and various cardiometabolic-health-related markers, mainly through short-term interventions (4–12 weeks) in overweight and obese individuals. It is worth noting that supervision by qualified instructors may play a key role in serving these populations when performing this mind–body exercise type in real-world conditions. Progressive Pilates training protocols seem to be an efficient and injury-free approach for individuals with unhealthy weight. In summary, evidence that emerged during the past decade suggested that supervised Pilates training protocols can be an efficient, safe, and attractive exercise mode for apparently healthy individuals affected only by overweightness or obesity. However, further randomized controlled trials with superior methodological quality implementing long-term interventions conducted in a free-living environment are warranted to examine the real-world impact of Pilates training on a wide-ranging spectrum of psychophysiological health markers in overweight and obese individuals of all ages. International guidelines on physical activity and exercise [14,68,91] should evaluate future findings on Pilates-based training programs adapted for individuals with unhealthy weight. Such an approach may update and adjust these recommendations to the masses impacted by one of the most challenging public health issues of the 21st century that also results in a heavy economic burden globally [3,92].

## Figures and Tables

**Table 1:** Data extracted from each article included for review.

Article (First Author, Year)	Country	Duration (Weeks)	Sample ^1^ N/F/M	Mean Age ± SD (Years)	Activity, BMI Classification	Study Design	Pilates Classification	Pilates Intervention Characteristics ^2^	Findings	Drop-Out ^3^
Cakmakçi (2011) [53]	Turkey	8	58/58/0	37.4 ± 9.8	Sedentary, Obese	Chronic ^4^, RCT	Mat/ Props	4 sessions/week, 60 min/session, supervised, field-based	BW, BMI, BF, WC, WHR (↓); LBM, RMR, flexibility (↑)	5%
Chaudhary (2020) [54]	India	10	30/0/30	30.0–45.0	Sedentary, Overweight	Chronic, RCT	Mat	5–6 sessions/week, 30–40 min/session, supervised, field-based	BF (↓)	0%
Chen (2020) [55]	China	16	39/39/0	N/A	Inactive, Obese	Chronic, RCT	Mat/ Props	3 sessions/week, 60 min/session, supervised, field-based	BW, BMI, BF (↓)	0%
Gorji (2015) [56]	Iran	8	30/30/0	41.1 ± 2.6	Sedentary, Overweight	Chronic, RCT	Mat	3 sessions/week, 60 min/session, supervised, field-based	BW, BMI, BF (↓); ADPN (↑)	0%
Gorji (2014) [57]	Iran	8	30/30/0	42.6 ± 3.9	Sedentary, Overweight	Chronic, RCT	Mat	3 sessions/week, 60 min/session, supervised, field-based	BW, BMI, BF, FetA (↓)	0%
Hagner- Derengowska (2015) [58]	Poland	10	108/108/0	58.7 ± 5.6	Inactive, Overweight/ Obese	Chronic, CT	Equipment	3 sessions/week, 60 min/session, semi-supervised, field-based	BW, BMI, TC, LDL, TG (↓); BG, HDL (↔)	28%
Jago (2006) [59]	United Kingdom	4	30/30/0	11.2 ± 0.6	Inactive, Overweight	Chronic, RCT	Mat	5 sessions/week, 60 min/session, supervised, field-based	BMI (↓); BW, WC, SBP, DBP (↔)	25%
Jung (2020) [50]	South Korea	12	32/32/0	47.5 ± 7.5	Inactive, Overweight/ Obese	Chronic, RCT	Mat/ Props	3 sessions/week, 50 min/session, supervised, lab-based	BW, BMI, SBP, DBP, MAP, BG, TC, TG (↓); BF, BG, BMD, BMC, HDL, LDL, HOMA-IR, INSL, VO_2max_ (↔)	11%
Khajehlandi (2018) [60]	Iran	12	28/28/0	29.9 ± 3.8	Inactive, Overweight	Chronic, CT	Mat/ Props	3 sessions/week, 50 min/session, supervised, lab-based, RPE~14	BM, BMI, WHR (↓); OCN (↑); PTH (↔)	0%
Khormizi (2017) [61]	Iran	8	30/30/0	51.9 ± 5.9	Sedentary, Obese	Chronic, RCT	Mat/ Props	3 sessions/week, 60 min/session, supervised, field-based	BW, BMI (↓)	0%
Savkin (2016) [62]	Turkey	8	37/37/0	43.8 ± 4.9	Sedentary, Overweight/ Obese	Chronic, RCT	Mat	3 sessions/week, 90 min/session, supervised, field-based, RPE~14	BW, BMI, BF, WC (↓); LBM (↔)	14%
Tyagi (2020) [63]	India	24	60/0/60	20.0–45.0	Inactive, Obese	Chronic, RCT	Mat	NR	BW, BMI, BF, VAT (↓)	NR
Vancini (2017) [64]	Brazil	8	42/33/9	31.5 ± 4.0	Inactive, Overweight/ Obese	Chronic, RCT	Mat/ Equipment	3 sessions/week, 60 min/session, supervised, field-based, 59% MHR	QoL (↑); DEP, ANX (↓)	13%
Wong (2020) [65]	United States	12	28/28/0	22.5 ± 4.0	Sedentary, Obese	Chronic, RCT	Mat	3 sessions/week, 60 min/session, supervised, field-based	BW, BMI, LBM (↔); BF, SBP, DBP, MAP (↓)	0%

ADPN, adiponectin; ANX, anxiety; BG, blood glucose; BF, body fat; BMC, bone mineral content; BMD, bone mineral density; BMI, body mass index; BW, body weight; CT, controlled trial; DBP, diastolic blood pressure; DEP, depression; FetA, Fetuin-A; HDL, high-density lipoprotein cholesterol; HOMA-IR, homeostatic model assessment for insulin resistance; INSL, insulin; LBM, lean body mass; LDL, low-density lipoprotein cholesterol; MAP, mean arterial pressure; MHR, maximum heart rate; NR, not reported; OCN, osteocalcin; PTH, Parathormone; TC, total cholesterol; TG, triglycerides; QoL, quality of life; RPE, rating of perceived exertion; RCT, randomized controlled trial; RMR, resting metabolic rate; SBP, systolic blood pressure; VAT, visceral adipose tissue; VO_2_max, maximal oxygen consumption; WC, waist circumference; WHR, waist-to-hip ratio. ^1^ Sample size refers to participants who completed (not being recruited) the study; ^2^ Session duration (including warm-up and cool-down); ^3^ Dropout rate refers to participants who did not complete the Pilates intervention; ^4^ Chronic responses (≥2 weeks). ↑ indicates higher; ↓ indicates lower; ↔ indicates unchanged.

## Supplementary Materials

The following supporting information can be downloaded at: https://www.mdpi.com/article/10.3390/diseases10040071/s1, Table S1: Search algorithms and results.
