# Supplementary material for: Psychophysiological Adaptations to Pilates Training in Overweight and Obese Individuals: A Topical Review
Source: Diseases. 2022 Sep 29;10(4):71. doi: 10.3390/diseases10040071 (PMC9589980; doi:10.3390/diseases10040071)
Supplement: Supplementary file 1 [file diseases-10-00071-s001.zip › diseases-1926402-supplementary.pdf]

Table S1. PubMed/MEDLINE search algorithms and results.

Search	Query	Items found
6	Search (4 AND 5)	21
5	Search pilates[Title/Abstract]	671
4	Search (1 OR 2 OR 3)	377,27
3	Search overweight[Title/Abstract]	82,457
2	Search obese[Title/Abstract]	142,951
1	Search obesity[Title/Abstract]	298,517
